# Growth dynamics of untreated meningiomas

**DOI:** 10.1093/noajnl/vdad157

**Published:** 2023-12-20

**Authors:** Per Sveino Strand, Kathrine Jørgensen Wågø, André Pedersen, Ingerid Reinertsen, Olivia Nälsund, Asgeir Store Jakola, David Bouget, Sayied Abdol Mohieb Hosainey, Lisa Millgård Sagberg, Johanna Vanel, Ole Solheim

**Affiliations:** Department of Neurosurgery, St. Olavs University Hospital, Trondheim, Norway; Department of Neuromedicine and Movement Science, Norwegian University of Science and Technology, Trondheim, Norway; Department of Pediatrics, St. Olavs University Hospital, Trondheim, Norway; Department of Health Research, SINTEF Digital, Trondheim, Norway; Department of Health Research, SINTEF Digital, Trondheim, Norway; Department of Circulation and Medical Imaging, Norwegian University of Science and Technology, Trondheim, Norway; Department of Clinical Neuroscience, Institute of Neuroscience and Physiology at the Sahlgrenska Academy, University of Gothenburg, Gothenburg, Sweden; Department of Neurosurgery, Sahlgrenska University Hospital, Gothenburg, Sweden; Department of Health Research, SINTEF Digital, Trondheim, Norway; Department of Neurosurgery, University Hospital Southampton, Southampton, UK; Department of Neurosurgery, St. Olavs University Hospital, Trondheim, Norway; Department of Public Health and Nursing, Norwegian University of Science and Technology, Trondheim, Norway; Department of Health Research, SINTEF Digital, Trondheim, Norway; Department of Neurosurgery, St. Olavs University Hospital, Trondheim, Norway; Department of Neuromedicine and Movement Science, Norwegian University of Science and Technology, Trondheim, Norway

**Keywords:** incidental meningiomas, Gompertzian, growth patterns, meningioma, tumor growth

## Abstract

**Background:**

Knowledge about meningioma growth characteristics is needed for developing biologically rational follow-up routines. In this study of untreated meningiomas followed with repeated magnetic resonance imaging (MRI) scans, we studied growth dynamics and explored potential factors associated with tumor growth.

**Methods:**

In a single-center cohort study, we included 235 adult patients with radiologically suspected intracranial meningioma and at least 3 MRI scans during follow-up. Tumors were segmented using an automatic algorithm from contrast-enhanced T1 series, and, if needed, manually corrected. Potential meningioma growth curves were statistically compared: linear, exponential, linear radial, or Gompertzian. Factors associated with growth were explored.

**Results:**

In 235 patients, 1394 MRI scans were carried out in the median 5-year observational period. Of the models tested, a Gompertzian growth curve best described growth dynamics of meningiomas on group level. 59% of the tumors grew, 27% remained stable, and 14% shrunk. Only 13 patients (5%) underwent surgery during the observational period and were excluded after surgery. Tumor size at the time of diagnosis, multifocality, and length of follow-up were associated with tumor growth, whereas age, sex, presence of peritumoral edema, and hyperintense T2-signal were not significant factors.

**Conclusions:**

Untreated meningiomas follow a Gompertzian growth curve, indicating that increasing and potentially doubling subsequent follow-up intervals between MRIs seems biologically reasonable, instead of fixed time intervals. Tumor size at diagnosis is the strongest predictor of future growth, indicating a potential for longer follow-up intervals for smaller tumors. Although most untreated meningiomas grow, few require surgery.

**Key Points**

- Untreated meningiomas followed with repeated magnetic resonance imaging (MRI) scans follow a Gompertzian growth curve.
- Increasing subsequent follow-up intervals between MRIs seems biologically reasonable, instead of fixed time intervals for patients with untreated meningioma.

**Importance of the Study**

Asymptomatic meningiomas are more often diagnosed than symptomatic meningiomas, and knowledge of how untreated meningiomas grow, and potential factors linked to tumor growth, is important for rational treatment and clinical follow-up of these patients. In this study, we find that untreated meningiomas follow a Gompertzian growth curve. This means that increasing and potentially doubling subsequent follow-up intervals between MRIs seems biologically reasonable, instead of fixed time intervals. Tumor size at diagnosis is the strongest predictor of future growth, indicating a potential for longer follow-up intervals in small tumors.

Meningioma is the most common intracranial tumor, with a reported prevalence of 0.9%–1.0% in population-based magnetic resonance imaging (MRI) studies.^1–4^ While surgery is usually the preferred primary treatment for symptomatic tumors, most meningiomas are now diagnosed in an asymptomatic phase.^5^ Management of incidental meningiomas varies, and if conservative management is chosen, routines for follow-up vary across guidelines and departments.^6^ The European Association of Neuro-Oncology (EANO) recommends annual MRIs the first 5 years after diagnosis, thereafter every 2 years.^7^

A systematic review found that most meningioma patients who experienced radiological tumor growth or clinical deterioration did so during the first 5 years from diagnosis.^6^ Further, a prospective study with 64 patients found that 75% of diagnosed meningiomas increased 15% or more in volume in 5 years, but no patients developed symptoms related to the tumor.^8^ In a long-term follow-up study of incidental meningiomas, 50% showed progression in 10 years and 75% exhibited growth over 15 years, suggesting that long-term follow-up may be necessary for many patients.^9^ Expected or potential pattern of meningioma growth could have implications for both frequency and length of follow-up. However, longer-term studies on growth dynamics in untreated patients are scarce, and exponential growth, linear growth, self-limiting growth, as well as spontaneous regression have been reported in previous studies.^8,10–14^ Assessment of growth of small tumors can be vulnerable to measurement errors, and automatic volumetric assessments may be beneficial to reduce intra- or interrater variability. Further, short follow-up may hamper the assessment of growth patterns. In this single-center volumetric study with automatic volume assessments of untreated meningiomas followed with repeated MRIs, we studied growth dynamics and explored potential factors associated with tumor growth. We sought to statistically compare different patterns of growth, including exponential growth, linear growth, linear radial growth, and Gompertzian growth, to assess goodness-of-fit on group level.

## Methods

### Inclusion Criteria

All adult patients (≥18 years) referred to the Department of Neurosurgery at St. Olavs University Hospital (Trondheim, Norway) with a radiologically suspected intracranial meningioma from 2006 to 2015 were screened for inclusion. The last follow-up was in August 2019. Only patients with 3 or more MRIs with at least a 6-month time interval between the scans were included. Patients who had been treated for a brain tumor previously had received radiation therapy to the brain, or were diagnosed with neurofibromatosis were excluded.

### MRIs and Clinical Data

Our department serves as a geographical catchment region for neurosurgery in mid-Norway, and MRIs were done in 10 different clinics in this region. Slice thickness varied from 0.5 to 6.6 mm. MRI scanner vendors included GE, Philips, Siemens, and Picker, with a total of 21 different scanner models. Patients, who were operated on during the observational period, were operated in general anesthesia with standard microsurgical techniques.

### Tumor Segmentation

Tumors were segmented using an automatic algorithm developed at our institution,^15^ using T1-weighted MRIs. After the initial automatic segmentation, all segmentations were controlled by one of the authors and, if needed, manually corrected using the software ITK-SNAP^16^ or 3D Slicer,^17^ based upon the preference of the evaluator. In patients with only noncontrast MRI series, tumors were manually segmented using T2-weighted images. If the patient harbored multiple meningiomas, the tumors were aligned with different labels which contributed to a single tumor volume.

### Statistical Analyses

Normality of data was determined using Kolmogorov–Smirnov tests. The data were not normally distributed and thus data were presented as medians and interquartile ranges (IQRs). Volumetric analysis of tumor size was based on automatic segmentations with, if needed, manual correction, and was measured in milliliters. Statistical significance level was set to 5%. Summary statistics were calculated in Python 3.7.9 using the SciPy library.^18^

### Growth Estimates and Assumptions for Growth Curves

Earlier studies have used a relative volume change of 15% as a cutoff for tumor growth.^10,19^ We analyzed margins of error of volume measurements based on reanalysis and manual segmentation of 20 random meningiomas from our cohort. Margins of error were defined using a bootstrapping method, rendering a median margin of error of 0.0895 (confidence interval [CI] 0.0651, 0.116). Based upon the similarity of the upper limit of the CI, and previous studies, a relative volume change of 15% was used as a cutoff for tumor growth or shrinkage in this study.

For meningioma growth curve modeling, it was hypothesized that the 4 statistical models described in Table 3 could be appropriate. These were linear, exponential, linear radial, and Gompertzian. Linear growth is when the tumor grows with a constant amount for each unit of time. In the exponential growth model, the tumor grows at a constant rate, whereas in the linear radial model, one assumes that the radius of the tumor follows a linear growth pattern, thus resulting in cubic growth of the tumor. Finally, in the Gompertzian growth model, one assumes the growth of the tumor first has an exponential growth phase, followed by a linear phase, and ultimately a plateau phase.

Tumor growth is a multilevel problem, as growth is estimated within the patient, but data also exist on population level. Hence, to perform the curve modeling, a nonlinear, multilevel, mixed-effect model approach was conducted. To perform the analysis, the menl library in Stata/MP 17.0 was used.^20,21^ The source code used to preprocess the tabular data and conduct the statistical analyses in both Python and Stata is made openly available at https://github.com/andreped/tumor-growth.

### Ethics

Ethical permission for the study was granted by the Regional Ethics Committee (REK reference 2016/1359). The data collection was done in line with the Declaration of Helsinki.

## Results

**Figure 1.**
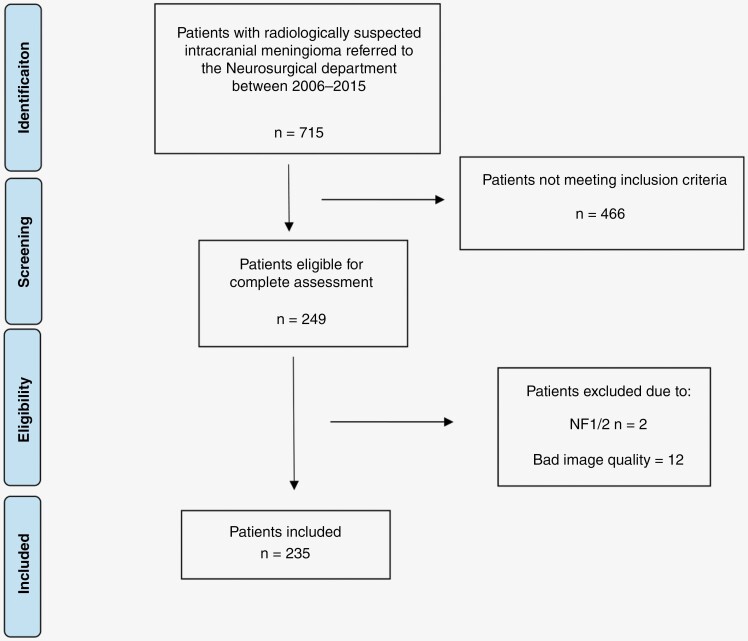
Flow chart of the inclusion process.

A flow chart of the inclusion progress is shown in Figure 1. In total, 235 patients were included for analysis. Patient characteristics are presented in Table 1. The median number of MRI observations was 5 (range 3–72), and the median tumor volume at diagnosis was 2.6 mL (range 0.1–64.2 mL). The median follow-up time in the study period was 63.0 months (range 7.2–188.3 months) and only 13 patients (5.5 %) underwent surgery during the follow-up period. Twenty-three patients had multiple meningiomas. In total, 138 of the 235 (58.7%) tumors grew during the observational period, whereas 33 (14.0%) tumors shrunk, and 64 (27.2%) tumors exhibited no radiological sign of growth.

**Table 1.** Patient characteristics

Number of patients	235
Number of scans	1394
Number of MRI scans per patient (median, IQR, range)	5 (3); 3–17
Age at diagnosis (median, IQR, range)	63.2 (16.3); 18.3–89.1
Tumor volume at diagnosis [ml] (median, IQR, range)	2.6 (4.8); 0.1–64.2
T2 signal	
Hypointense	137 [75.7%]
Isodense	31 [17.1%]
Hyperdense	13 [7.2%]
Peritumoral edema	
Yes	22 [10.1%]
No	196 [90.0%]
Tumor volume change (±15%)	
Growth	138 [58.7%]
No change	64 [27.2%]
Shrinkage	33 [14.0%]
Multifocality (number, percentage)	23 [9.8%]
Total follow-up period [months] (median, IQR, range)	63.0 (55.9); 7.2–188.3

A total of 1394 MRI scans were carried out in the 235 patients in the median 5-year observation period. The median volume change in the observation period was 0.5 (1.6; −24.5 to 76.7) mL, and the median relative change was 24.6 (62.4; −90.9 to 153.4)%. Thirteen patients (5.5%) who all harbored growing tumors ended up with surgical treatment in the observation period. Only MRI data before surgical treatment were assessed. MRI characteristics are presented in Table 2. Variations in slice thickness and/or variations in magnetic strength over time were not linked to measured tumor growth or shrinkage.

**Table 2.** MRI characteristics

Slice thickness in millimeters (median, IQR, range	1.0 (5.1); 0.5–6.6
MRI scanner vendor	*N* = 1394 scans
General Electric (GE)	191 [13.7%]
Philips	576 [42.0%]
Siemens	587 [42.1%]
Picker	24 [1.7%]
*Missing*	6 [0.4%]
Magnetic field strength [Tesla]	*N* = 1394 scans
1.0	14 [1.0%]
1.5	887 [63.6%]
3.0	487 [34.9%]
*Missing*	6 [0.4%]

### Which Growth Pattern Characterizes Meningiomas?

Four different statistical models were compared to find the best-suited regression model for explaining meningioma growth at group level. The mathematical descriptions of the statistical models and the goodness-of-fit for the different regression models are presented in Tables 3 and 4, respectively. The exponential, linear, radial, and Gompertzian functions were vastly superior to the linear model, in terms of log likelihood, Akaike Information Criterion, and Schwarz’s Bayesian Information Criterion. The Gompertzian model was the best-suited model overall to characterize meningioma growth.

**Table 3.** Mathematical description of the statistical models tested

Model	Mathematical model	Statistical model (model tested)
Linear	V(t)=βt+α	V(t)=βt+α+ϵ=μ+ϵ
Exponential	$V\left(t\right)=V_{0}*e^{\alpha t}$	$\log\left(V\left(t\right)\right)=\log\left(V_{0}\right)+\alpha t+\epsilon =\mu +\epsilon$
Linear radial	$V\left(t\right)=\frac{4\pi }{3}*{\left(r_{0}+\alpha t\right)}^{3}$	$\log\left(V\left(t\right)\right)=\log\left(\frac{4\pi }{3}\right)+3*\log\left(r_{0}+\alpha t\right)+\epsilon =\mu +\epsilon$
Gompertzian	$V\left(t\right)=K*e^{\log\left(\frac{V_{0}}{K}*e^{-\alpha t}\right)}$	$\log\left(V\left(t\right)\right)=\log\left(K\right)+\log\left(\frac{V_{0}}{K}\right)*e^{-\alpha t}+\epsilon =\mu +\epsilon$

**Table 4.** Goodness-of-fit for the different regression models

Model	Log likelihood	AIC	BIC
Linear	−3445.5	6913.1	6969.1
Exponential	−525.8	1073.7	1128.8
Linear radial	−469.5	961.0	1016.0
Gompertzian	−407.5	839.0	899.1

### Factors Associated With Growth

Potential factors associated with tumor growth measures are presented in Table 5. As seen, larger tumors at diagnosis, longer duration of follow-up, and multifocality were associated with growth, while age, sex, T2 signal, and presence of edema were not. A separate model was constructed where patients with multifocal tumors were excluded, which rendered the same results as in the model above.

**Table 5.** Association between tumor growth and risk factors using the Gompertzian model

Variable	Coefficient	Standard error	*P*-value	95% CI
Sex	0.065	0.041	0.115	[−0.158, 0.146]
Age at first scan	0.001	0.001	0.462	[−0.001, 0.003]
Follow-up in months	0.001	<0.001	<0.001	[0.001, 0.002]
Initial tumor volume	0.219	0.017	<0.001	[0.187, 0.252]
Multifocality	0.147	0.087	0.020	[0.024, 0.279]
T2 hyperintensity Hypo- vs Isodense Hypo- vs Hyperdense	−0.098 −0.044	0.083 0.081	0.239 0.584	[−0.262, 0.065] [−0.203, 0.114]
Cerebral edema	0.147	0.087	0.090	[−0.023, 0.317]

## Discussion

In this cohort study of untreated meningiomas followed with repeated MRIs, we found that 58.7% grew, 27.2% remained stable, and 14.0% shrunk in measured volume over the median observation period of more than 5 years. A Gompertzian growth curve best describes the growth dynamics at group level. Tumor size at diagnosis, multifocality, and length of follow-up were factors associated with tumor growth. Age, sex, the presence of edema, and T2 hyperintensity were not significantly associated with growth in the present study.

There is no standardized definition of meningioma growth in previous studies,^22,23^ making comparison of results across studies difficult. Also, depending on growth pattern, different metrics for evaluating tumor growth rate can be appropriate. For tumors showing linear growth, annual growth rate or annual volume change would be the more appropriate measure, while tumors with exponential growth should be measured using relative growth rate.^24^ Still, from a biological point of view, linear growth seems unlikely.

While a range of growth patterns have been reported in meningioma, 1 previous study reported that benign meningiomas follow a Gompertzian growth curve.^12^ Gompertzian tumor growth is biologically plausible and reported in several other tumor entities than meningioma.^25,26^ A Gompertzian growth curve, which has an early exponential phase, followed by a linear-like phase and eventually a plateau phase could perhaps explain the variations in reported growth patterns as studies never capture the entire natural course of meningioma. Follow-up time may affect observed growth patterns and it has previously been found that follow-up times for meningiomas exhibiting self-limiting growth are significantly longer than for tumors with linear/exponential growth.^8^ The presence of calcifications could indicate the plateau phase.^27^ Further, it is possible that small-size meningiomas are more likely to be subject to relatively larger errors of measurement that may overshadow true growth.^27^ Automatic volume assessments, as done in the present study, may reduce the problem of inter-and intrarater variability that can be problematic, especially in the assessment of small volumes. Given a Gompertzian growth pattern, tumor doubling times will be longer and not shorter over time, justifying a doubling of intervals between each follow-up MRI unless growth is detected, instead of fixed intervals. In light of this, the consensus-based current guidelines from the European Association of Neuro-Oncology (EANO) that recommend annual MRIs for 5 years after diagnosis, and thereafter active follow-up with intervals of 2 years, could be debated.^7^

Although frequent MRIs make it possible to detect asymptomatic tumor growth at an early stage, excessive imaging is costly, may result in patient anxiety,^9,28^ and only a minority of incidental meningiomas may require treatment.^29^ Detected growth may be used as an indication for treatment; however, as meningioma exhibits Gompertzian growth patterns, current growth does not necessarily predict the future growth of a meningioma.

In our cohort, 56.8% of the tumors displayed growth during observation, and the results are in line with other studies.^22,23^ However, as seen in the present study, only 5.5% of the tumors ended up being treated even if a majority grew. Also, 1394 scans were carried out to detect growth in the 13 asymptomatic patients who ended up with surgery. Since a majority of asymptomatic meningiomas will exhibit growth within 5 years, one may argue that upfront stereotactic radiotherapy may be an attractive option, in accordance with the IMPASSE study.^30^ However, such an active approach could potentially result in overtreatment of many patients. A meningioma of a certain size has grown at some point, even if it was not documented with repeated MRIs at the time of growth. Further, in our study, almost one-third of tumors are stable during follow-up, and 14.0% of tumors shrunk, indicating that detecting growth in a given period of time in the asymptomatic phase not necessarily is a predictor of future growth. Given the described Gompertzian growth pattern, a decrease in growth rates over time may be expected in many patients.

A more fundamental question is if measured growth is clinically relevant. Are treatment outcomes significantly worse if we wait? Surprisingly, 1 study found that patients who were operated for asymptomatic meningiomas experienced more complications from surgery than patients who were operated for symptomatic meningiomas.^31^ Further, a recent matched case–control study found that there was no difference in functional or clinical outcomes between patients operated on for symptomatic versus incidental meningiomas.^32^ Although early surgery for incidental meningiomas can be indicated for patients with suspected higher histopathological grade (i.e. peritumoral edema, or invasive and rapid growth), or in patients where an attractive treatment window for stereotactic radiotherapy (e.g. <3 cm) may be lost, or patients where the tumor is gradually approaching cranial nerves or vessels that would make later surgery more difficult or risky, one may in light of the often good functional outcome in many symptomatic meningioma patients argue that monitoring until symptoms could be a viable option in many patients.

A rational monitoring algorithm requires knowledge of the natural course to detect outliers that require special surveillance or treatment. As mentioned above, measuring growth only (yes/no) can be of unclear clinical value, and to no surprise, follow-up time is a predictor of growth. In line with most, but not all studies,^8,33,34^ we found a significant association between the initial tumor volume and tumor growth. This may be explained by more tumor cells prone to division and thus increased growth. Additionally, larger tumor volumes could be a marker of previous growth and previous growth could be a predictor of future growth potential. Also, the error of measurement relative to the tumor volume is smaller in large tumors, making it easier to detect growth. If accepting Gompertzian growth on group level and that time and size are the two most important predictors of growth, there may be a potential for developing normative growth curves, like for head circumference or height in children, to detect crossing of percentiles and tailor follow-up for individual patients. However, large datasets are needed to construct such nomograms, and as manual segmentation can be very time-consuming, automatic volumetric assessments would greatly enhance such an initiative. We have previously developed a rather accurate automatic algorithm for meningioma segmentation,^14^ but the biggest inaccuracies are seen in both manual and automatic segmentation of small tumors. The start and stop of the dural tail are not easily defined on MRIs and the chosen cutoff may have a significant contribution to the assessment of smaller tumor volumes.

### Strengths and Limitations

The strengths of the present study are the relatively large sample size and several observations over time for each patient. We used tumor segmentation for volume measurements and assessed errors of measurement. Moreover, the population-based referral minimizes selection bias in this study of incidental meningiomas.

A 15% change in tumor volume has been used to define growth in 2 previous studies,^10,19^ and for the present study, a similar 15% volume change was set as a cutoff to determine tumor growth or shrinkage. This may seem a somewhat conservative estimate considering our margins of error measurements. Still, as we only did repeated measurements in 20 of the patients, we decided to use the more conservative cutoffs. However, it may be argued that the cutoff value is too conservative, and in tumors with very small absolute volumes (the median tumor volume at the time of diagnosis was 2.6 mL), a small measurement error might result in a large difference in percentages.

However, follow-up MRI scans were done at a range of local hospitals where there is a large variation in slice thickness and MRI scanners. This may introduce a bias to our results, as higher slice thickness may lead to more uncertainty in the volume estimates. Still, variations in slice thickness and/or variations in magnetic strength over time were not linked to measured tumor growth or shrinkage in our data. There is no consensus on the slice thickness of meningioma follow-up imaging, but as several diagnostic scans were performed with low-resolution MRI scans, whereas follow-up scans were done with high-resolution scans, this could possibly have resulted in an overestimation of the initial tumor volume.^35^

It is possible that factors not included in our analyses affect the growth dynamics of meningiomas, for instance, menopausal status, the use of contraceptives, pregnancies, and genetic factors. Further, it is impossible to detect *when* a meningioma started to grow, and thus not possible to establish the exact growth patterns of meningiomas based on repeated tumor segmentations over time.

## Conclusions

In this single-center cohort study of untreated meningiomas followed with repeated MRIs, we found that 58.7% of the tumors grew, 27.2% remained stable, and 14.0% shrunk in volume over the median observation period of more than 5 years. Only 5.5% needed surgery during the observational period. Tumor size at diagnosis, multifocality, and length of follow-up was associated with tumor growth. A Gompertzian growth curve best describes growth dynamics on group level, indicating that doubling of follow-up MRI intervals over time may be more rational than fixed intervals in the surveillance of incidental meningiomas.

## Data Availability

The data used for this study will be made available upon reasonable request.
